# Multiply Charged Helium Droplet Anions

**DOI:** 10.1002/chem.202005004

**Published:** 2021-03-12

**Authors:** Felix Laimer, Fabio Zappa, Paul Scheier, Michael Gatchell

**Affiliations:** ^1^ Institut für Ionenphysik und Angewandte Physik Universität Innsbruck Technikerstr. 25 6020 Innsbruck Austria; ^2^ Departamento de Física-ICE Universidade Federal de Juiz de Fora Campus Universitário 36036-900 Juiz de Fora MG Brazil; ^3^ Department of Physics Stockholm University 10691 Stockholm Sweden

**Keywords:** charge carriers, helium droplets, multiple charges, superfluid

## Abstract

The detection of multiply charged helium droplet anions is reported for the first time. By ionizing droplets of superfluid helium with low energy electrons (up to 25 eV), it was possible to produce droplets containing up to five negative charges, which remain intact on the timescale of the experiment. The appearance sizes for different charge states are determined and are found to be orders of magnitude larger than for the equivalent cationic droplets, starting at 4 million He atoms for dianions. Droplets with He*^−^ as charge carriers show signs of being metastable, but this effect is quenched by the pickup of water molecules.

## Graphical abstract

**Charged droplets**: The detection of multiply charged helium droplet anions is reported for the first time. By ionizing droplets of superfluid helium with low energy electrons (up to 25 eV), it was possible to produce droplets containing up to five negative charges, which remain intact on the timescale of the experiment.

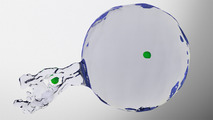

## Introduction

For more than two decades, the highest charge state ever reported in an isolated droplet of liquid helium was two.^[1]^ In 1997, Fárník et al.^[1]^ performed a detailed study of positively and negatively charged droplets of superfluid helium and identified a critical size of about 2×10^5^ He atoms before droplets could contain two positive charges. But in that study no higher charge states were observed and no droplets containing multiple *negative* charges could be detected. The possibility of higher charge states in helium droplets was not obvious given the very low binding energies (about 0.06 meV per atom) and other unique characteristics of the liquid. ^4^He droplets formed in vacuum have an equilibrium temperature of 0.37 K and are in a superfluid state, which results in an environment within the droplets with no internal friction and an extremely high thermal conductivity. Helium droplets are capable of capturing and solvating a wide range of atomic and molecular species, which, together with their aforementioned properties, makes them a useful tool for experimental studies in, for instance, chemistry and chemical physics.[^2^, ^3^, ^4^] Examples of processes that have been studied by using doped He droplets are reaction products of metal nanoparticles and organic molecules,^[5]^ reactions between atomic radicals and complex molecules,^[6]^ and sub‐Kelvin proton transfer reactions.^[7]^ Helium droplets are also a versatile matrix for photochemical and spectroscopic studies of cold molecules and ions.[^2^, ^8^, ^9^, ^10^, ^11^, ^12^]

Recently, it was demonstrated that He droplets can indeed hold multiple positive charges.^[13]^ In that study, a novel setup was used where neutral droplets containing millions of He atoms each were ionized by impact of energetic electrons, mass‐per‐charge selected by using an electrostatic analyzer, and then re‐ionized by using a second identical ion source before the final products were analyzed by using a second set of electrostatic deflectors. Using the second ionizer and analyzer, multiply charged precursor droplets selected in the first stage could be identified by increasing or decreasing their net charge states. This led to the appearance of narrow product peaks in the recorded mass spectra with mass‐per‐charge ratios at distinct fractions of the position of the precursor peak, with the fractions being equal to the ratio of the precursor charge over the product charge states. The smoking gun proving that this was actually from charge buildup in the droplets and not from symmetric Coulomb explosions was that the charge in the precursor droplets could be decreased, giving product droplets with *higher* mass‐per‐charge ratios than the precursors.^[13]^ Net charge states up to 55+ were identified and the critical droplet size as a function of charge states was measured,^[13]^ revealing critical sizes smaller than previously found by Fárník et al. for dications.^[1]^ In comparison, there have been no reported measurements of droplets containing more than a single net negative charge despite attempts to produce them.^[1]^

From a purely electrostatic picture and for a given charge distribution, there is no reason why the number of negative charges that a droplet could contain before the repulsive Coulomb forces overcome the cohesive forces of the liquid would be any different from the number of positive charges. However, negative charge carriers in pristine liquid He are chemically very different than their cationic counterparts. Positive charge carriers in He droplets are, after the initial ionization processes, predominantly He^+^ ions, which readily form covalently bound molecular dimers or trimers together with neighboring atoms.[^14^, ^15^] These ions can remain solvated in the droplets and are expected to form the cores of densely packed clusters that are known as Atkins snowballs.^[16]^ Negative charge carriers on the other hand consist of either electrons or He*^−^ ions^[17]^ (isolated He anions are metastable with a lifetime of 359 μs[^18^, ^19^]). Both face a strong, short‐range repulsive potential owing to the Pauli repulsion with the 1 s electrons in the surrounding neutral He atoms and thus form voids in the liquid that are expected to migrate towards the surface.^[17]^ This heliophobic nature causes negative charge carriers to be more easily expelled from pristine He droplets, especially if more than one are present. However, it has been predicted that large enough droplets could allow multiple negative charge carriers to remain long enough to be detected on experimental timescales.^[1]^

In this paper, we report the first (to our knowledge) detection of He droplets containing multiple negative charges. These were produced by using the same setup as was used to study droplets containing multiple positive charges in ref. [13]. Neutral droplets of superfluid He were produced by supersonic expansion of compressed He from a cryogenically cooled nozzle. The droplets were studied by using a tandem setup consisting of an electron impact ion source and electrostatic analyzer, followed by a second identical ionizer and analyzer. Impacting the droplets by using electrons with energies close to or below the ionization threshold of He, negatively charged droplets were formed. The two‐stage setup of our apparatus allowed us to select droplets after the first ion source based on their mass‐per‐charge ratios for additional probing by using the second ion source.

## Results and Discussion

**Figure 1:**
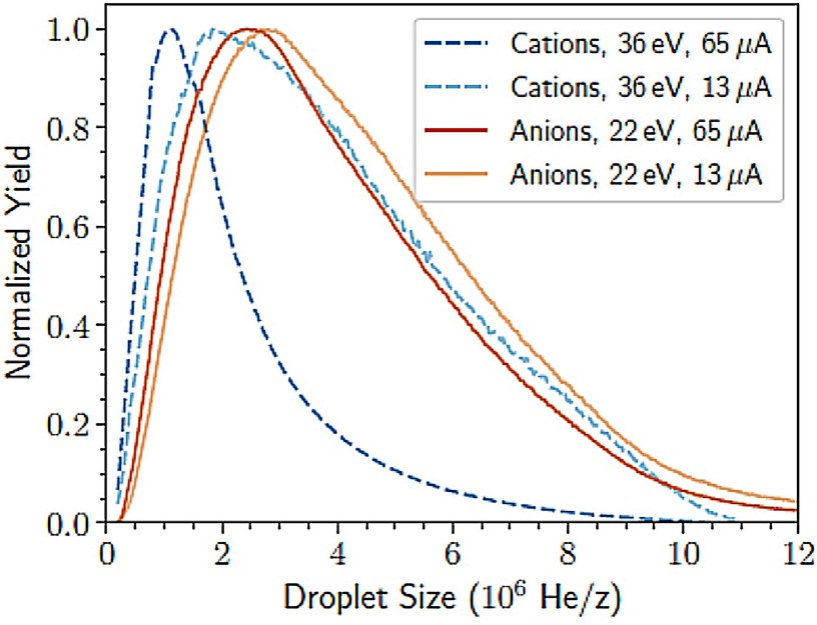
Mass‐per‐charge distributions of He droplets, produced with a nozzle temperature of 8 K and backing pressure of 20 bar, ionized with 36 eV (cationic droplets, dashed blue) and 22 eV (anionic droplets, solid red) electrons.

Helium droplets produced under the conditions used here, with nozzle temperatures in the range of 6 to 8 K and a 20 bar backing pressure, have a broad log‐normal size distribution with sizes in the range of 10^6^–10^8^ He atoms. The exact neutral distribution can, however, not be directly measured with the present setup. As was previously noted for cations,^[13]^ when the droplets are ionized, the buildup of charge causes the apparent size distribution (in units of mass per charge) to be shifted to lower values, an effect that is correlated with higher ionization energies and currents. For anions under otherwise identical conditions, this effect is much weaker, as seen in Figure 1. With 22 eV electrons, increasing the ionization current from 13 to 65 μA has only a small effect on the measured size distribution of negatively charged droplets, which in addition to charge buildup could also be caused by the evaporation of He as the droplets are heated by the impinging electrons. These findings indicate that there is less of a charge buildup with negative charge carriers than for cations.

**Figure 2:**
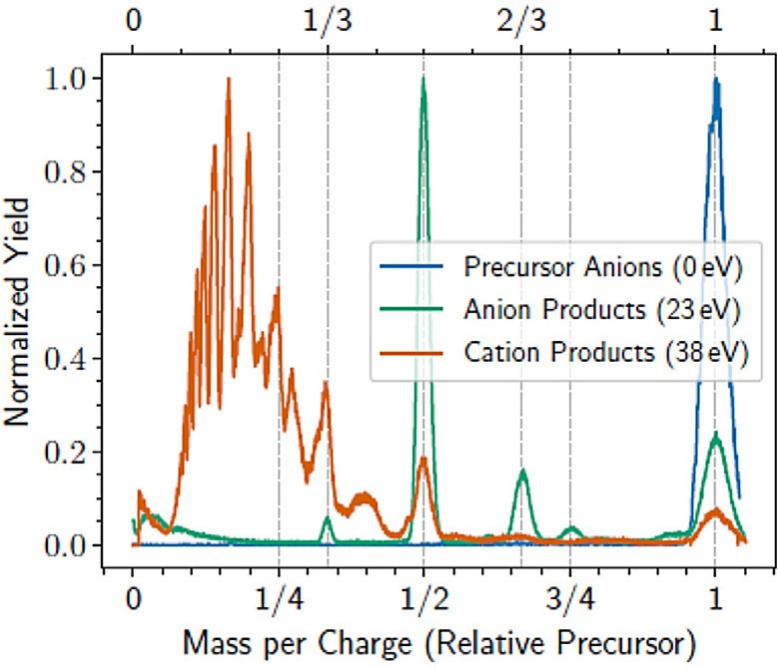
Negatively charged droplets containing 2.49×10^7^ He per charge were selected in the first stage after being ionized by 25.9 eV electrons. Product distributions were measured for three different settings on the second ion source: 0 eV (second source off, in blue), anionic products with 23 eV electrons (green), and cationic products with 38 eV electrons (red). Peak positions correspond to the ratios $\left|z_{1}/z_{2}\right|$ .

The same effect can also be seen in Figure 2 where we have selected a single precursor mass‐per‐charge ratio of negatively charged droplets with the first analyzer. In these measurements, anionic droplets consisting of 2.49×10^7^ He atoms per charge were selected following ionization by 25.9 eV electrons. Using 38 eV electrons in the second ion source and scanning through the cationic products (flipping the polarity of the second analyzer), we obtained the red data in Figure 2. A series of peaks occurring at rational fractions of the precursor mass‐per‐charge ratio are seen from products containing multiple net positive charges. The fractions are determined by the absolute value of the ratio of charge states after the first and second analyzers, $\left|z_{1}/z_{2}\right|$ . The series of peaks at values of 1/*n* of the size of the precursor droplets can be resolved for *n* up to about 10 and weaker peaks originating from doubly charged precursors are visible as well (for instance, the peaks at positions 2/3 and 2/5). Starting with the same precursors, the anionic products only display charge states up to 3− from singly charged precursors (peak at position 1/3), whereas charge states up to 4− can be identified as originating from triply charged precursors (peak at 3/4). Comparing the anion and cation products in Figure 2, it is immediately clear that the number of charges present in the anions is much lower than for the cations. The widths of the peaks are, however, similar and in both cases with a relative FWHM of about 5 %.

**Figure 3:**
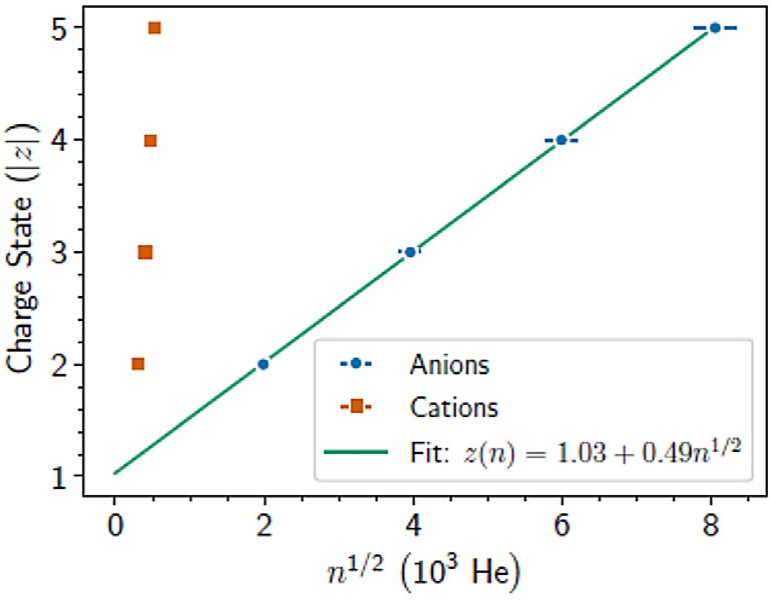
Measured critical sizes of He_*n*_^*z*^ droplets for charge states up to *z=*5± (cation data from ref. [13]). The appearance sizes for anions are significantly larger than for cations and the negative charge that can be held by a droplet apparently scales with √*n*.

By scanning the two sets of analyzers in tandem, we have determined the critical sizes for negatively charged He droplets for charges up to *z=*5−. The results of these measurements are shown in Figure 3 and Table 1 together with results for cationic droplets.^[13]^ Compared with the cationic droplets, the appearance sizes for multiply charged anionic droplets are some two orders of magnitude larger. The critical size for dianions, approximately 4 million He atoms, is an order of magnitude smaller than the theoretically predicted appearance size (3×10^7^ He) of metastable He droplet anions, which survive on millisecond timescales.^[1]^ It is also only slightly smaller than the measured appearance size for positively charge droplets containing 35 charges.^[13]^ Furthermore, the trend to larger sizes is distinctly different between positive and negative charge states. For cations, the number of charges that can be contained in a stable droplet scales with the surface area (assuming spherical droplets), indicating that the charges occupy the surface of the liquid.^[13]^ For anions, the charge stability appears to scale with the square root of the number of He atoms (or volume) for the charge states studied here. The least square fit to the measurements is shown in the legend of Figure 3 and from this curve we deduce a simple empirical scaling law for the negatively charged droplets where the critical size in units of 10^6^ He atoms, *n*, for a given negative charge state, *z* (the absolute value), to a good approximation is given by Eq. 1

(1) $$n\left(z\right)\approx 4(z-1{)}^{2}$$

**Table 1:** Measured appearance sizes for multiply charged anionic and cationic He droplets. Cation data from ref. [13].

*z*	*n*_crit_^−^	*n*_crit_^+[13]^
2±	(3.95±0.20)×10^6^	(1.00±0.05)×10^5^
3±	(1.57±0.8)×10^7^	(1.63±0.08)×10^5^
4±	(3.59±0.18)×10^7^	(2.17±0.11)×10^5^
5±	(6.49±0.32)×10^7^	(2.71±0.14)×10^5^

The origin for this type of scaling is not immediately clear. The quadratic relationship between appearance size and charge is consistent with classical liquid drop models,^[20]^ but contrasts with the scaling seen for cationic droplets.^[13]^ One factor that comes into play for the anions, but is absent for cations, is that negatively charged droplets are expected to be metastable. The measured critical size would then be dependent on the measurement timescale and larger droplets containing multiple negative charge centers could be detected simply owing to the longer time it takes for charges to escape the droplets. However, in measurements where we vary the flight time by up to 25 % (by adjusting the nozzle temperature[^21^, ^22^]), we see no measurable difference. Additional time‐dependent measurements or advanced modeling would thus be required to investigate this and lies beyond the scope of this work.

**Figure 4:**
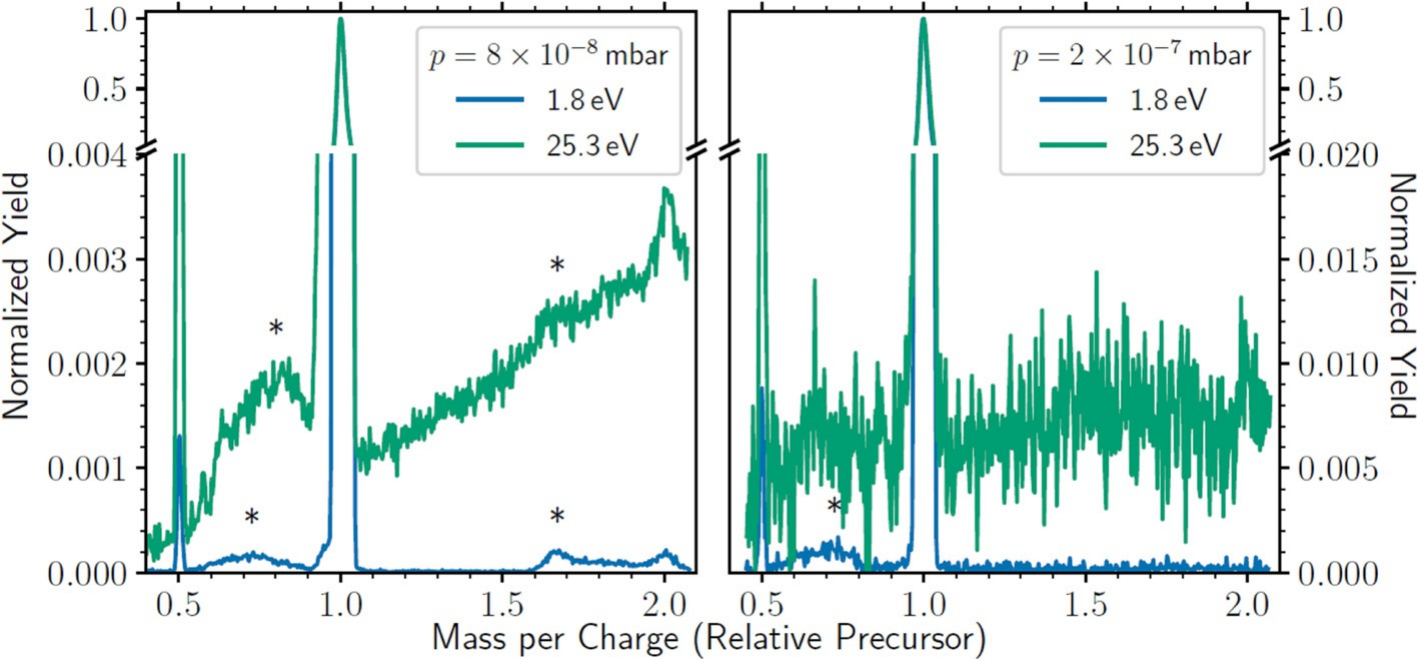
Scans of selected droplet with 4.5×10^6^ He atoms per negative charge ionized a second time with 1.8 eV or 25.3 eV electrons for two different chamber pressures. The residual gas consists mainly of water and the higher pressure increases the yield of doped droplets, which has a stabilizing effect on the negative charges, most notable for the He*^−^ ions (green curves). Features indicated with asterisks (*) originate from photon emission as charged droplets strike the walls of the analyzer, see the Supporting Information for more details.

For the results thus far, we have not discussed what the negative charge carriers consist of. The reason for this is that we see no distinguishable difference in the critical sizes of anionic droplets where electrons or He*^−^ ions are the charge carriers. It is, however, possible to introduce dopants that form heliophilic charge carriers, which energetically favor being solvated in liquid He. Residual water will always be present in small amounts, even under optimal vacuum conditions, and the geometrical cross section of a droplet containing many millions of atoms is large. Captured water molecules may form clusters in the cold droplets that are heliophilic even as anions and are thus more likely to remain submerged. In Figure 4, we show scans of droplets initially ionized in the first ion source with 25.9 eV electrons, filtered with a mass per charge of 4.5×10^6^ He atoms per charge, and ionized a second time with 1.8 eV or 25.3 eV electrons in the second ion source. In the left panel, the measurements were performed under nominal conditions and in the right panel the same measurements were performed while baking the apparatus to roughly 100 °C, which increased the background pressure (mostly originating from stagnant He gas) from 8×10^−8^ mbar to 2×10^−7^ mbar. Based on previous measurements with a similar experimental setup,^[17]^ we estimate that the fraction of droplets containing one or more water molecules per charge to be approximately 5 % under our nominal conditions. When the apparatus is heated, we instead expect several water molecules per charge, on average, to be captured by the droplet beam.

In the lower pressure conditions, the precursor peak is visible at a relative mass per charge of one. At half and twice this value, products are visible with double or half of the precursor charge state, respectively, for both electron energies. However, when increasing the pressure, the peak twice the precursor mass per charge is absent at both energies. At the higher electron energy (25.3 eV), there is also a (weak) continuously increasing signal towards higher mass per charges, which is only present at lower pressures. This feature appears to originate from metastable droplets that decay in the second analyzer. If the decay takes place through the ejection of a charge center with little associated mass loss, then for every voltage on the deflector plates there will still exist a trajectory where droplets that lose a charge in the analyzer may reach the detector. Droplets detected at a relative mass per charge just above one will reach the detector if they eject a charge shortly before the end of the analyzer and droplets detected at a higher relative mass per charge will have lost a charge further into the analyzer. In addition to these features, there are humps labeled with asterisks (*) that result from photon emission as charged droplets strike the walls of the second analyzer. This type of emission has been previously reported for He cations,[^23^, ^24^, ^25^, ^26^, ^27^] but this is to our knowledge the first time it has been observed with anions. These are discussed further in the Supporting Information.

The absence of continuous decay of metastable droplets in the electrostatic analyzer in the conditions with higher residual pressure indicates that the droplets are stabilized by the capture of water. This is also supported by the lack of a peak at a relative mass per charge of two while the precursor peak and peak from droplets with an increased charge remain intact. These results also indicate that isolated electrons are more stable charge carriers than He*^−^ ions as no continuous decay is present for the prior.

## Conclusion

We have identified droplets of superfluid He containing up to five negative charges. The appearance sizes for a given charge state are orders of magnitude larger than for the equivalent cations and the scaling differs as well, with the anion scaling with the square root of the droplet size. The appearance size does not appear to depend on the species of charge carrier, either electrons or He*^−^, but the latter displays a weak, continuous decay in our analyzer, which is quenched by the pickup of residual water molecules. These findings are important for our understanding of this exotic liquid, in particular in experiments where (doped) droplets are ionized. The recent discovery of multiply charged cationic He droplets has already led to the development of new experimental techniques that take advantage of such systems. The benefits of using charged over neutral He droplets includes greater control over the sizes of clusters formed in doped droplets and new techniques for soft ionization.[^7^, ^28^] We foresee similar developments that use anionic droplets, in particular in ways that take advantage of the lower degree of charge buildup in these species, which leads to a greater degree of control over absolute droplet size and charge state than for cations.

## Experimental Section

The measurements were performed by using the apparatus described in ref. [13] and the Supporting Information of that paper. Droplets of superfluid liquid helium are produced by expanding compressed (backing pressure of 20 bar) and pre‐cooled He gas through a 5 μm nozzle on a copper block mounted to the second stage of a closed cycle cryo‐cooler. Resistive heating is used to control the temperature of the nozzle, which is typically operated in the range of 4 to 10 K. The neutral beam of He droplets passes through a 0.8 mm skimmer located 8 mm downstream from the nozzle before it is ionized by electron impact. Charged droplets are analyzed by using a 90° spherical electrostatic analyzer with a central radius of 7 cm. Following this is an identical ion source and electrostatic analyzer, which is used to re‐ionize and study the mass‐per‐charge selected droplets passing through the first analyzer. The products are detected by using a channel electron multiplier detector located after the second analyzer. The entire apparatus operates at room temperature with a vacuum pressure in the 10^−9^ mbar range. The mass scales of the measurements are calibrated by using measurements of He droplet velocities by Henne and Toennies.[^21^, ^22^]

## Conflict of interest

The authors declare no conflict of interest.

## Supporting information

As a service to our authors and readers, this journal provides supporting information supplied by the authors. Such materials are peer reviewed and may be re‐organized for online delivery, but are not copy‐edited or typeset. Technical support issues arising from supporting information (other than missing files) should be addressed to the authors.

Supplementary
