# Supplementary material for: Multiply Charged Helium Droplet Anions
Source: Chemistry. 2021 Mar 12;27(25):7283–7. doi: 10.1002/chem.202005004 (PMC8251920; doi:10.1002/chem.202005004)
Supplement: Supplementary file 1 — Supplementary [file CHEM-27-7283-s001.pdf]

Chemistry–A European Journal

Supporting Information

Multiply Charged Helium Droplet Anions

Felix Laimer,[a] Fabio Zappa,[a, b] Paul Scheier,[a] and Michael Gatchell*[a, c]

Author Contributions

F.L. Data curation: Equal; Formal analysis: Equal; Investigation: Lead; Writing – review & editing: Supporting
F.Z. Investigation: Supporting; Supervision: Supporting; Writing – review & editing: Supporting
P.S. Conceptualization: Lead; Funding acquisition: Lead; Supervision: Lead; Writing – review & editing: Supporting
M.G. Data curation: Equal; Formal analysis: Supporting; Funding acquisition: Supporting; Supervision: Supporting; Visualization: Lead; Writing – original draft: Lead; Writing – review & editing: Lead.

Apart from being widely employed as detectors for ions and electrons, channel electron multipliers can also be used to detect incident ultraviolet photons[1]. Collisions of ions on metal surfaces have shown to be a source of both broadband and narrow line ultraviolet photon emission[2]. Also for He ions this effect has been observed previously in various metals[3-5]. In our experiment, such a collision between He ions and the metal surface of the analyzer's sector plates is expected to occur during a scanning process. To be able to distinguish features originating from photon emission and true ion signal, we installed a deflector plate parallel to the He ion beam in between the exit orifice of the second analyzer and the opening of the channel electron multiplier detector, as shown in Figure 2. By applying a potential to this deflector plate, charged helium droplets can be prevented from entering the detector opening, which results in an attenuation of ion signal. Figure 1 shows three scans of selected precursor droplets at varying deflection potentials. The measurements show an attenuation in signal of the precursor and the peak with twice the precursor mass with rising deflection potential. As the feature marked with an asterisk does not show a similar trend and therefore seems to be unafflicted by the electric field of the deflector, we assign this feature to ion induced photon emission by the charged droplet beam colliding with the outer sector plate during the scanning process. Another indication that this feature is generated by the charged droplet beam hitting the analyzer plate is that we observe the relative position of this feature and the precursor droplet to be constant upon changing precursor size. Since the analyzer exit is embodied by a small orifice, we assume that there has to be a direct line of sight from the point where the droplet beam hits the outer analyzer plate and the channel electron multiplier for the emitted photons to be detected. As our measurements showed, the relation between the analyzer voltage that is needed for the beam to be bent to this point and the analyzer voltage that enables the droplet beam to reach the detector is constant. This constant relation of analyzer voltages directly reflects in a constant mass per charge relation between the treated feature and the precursor that we observed.

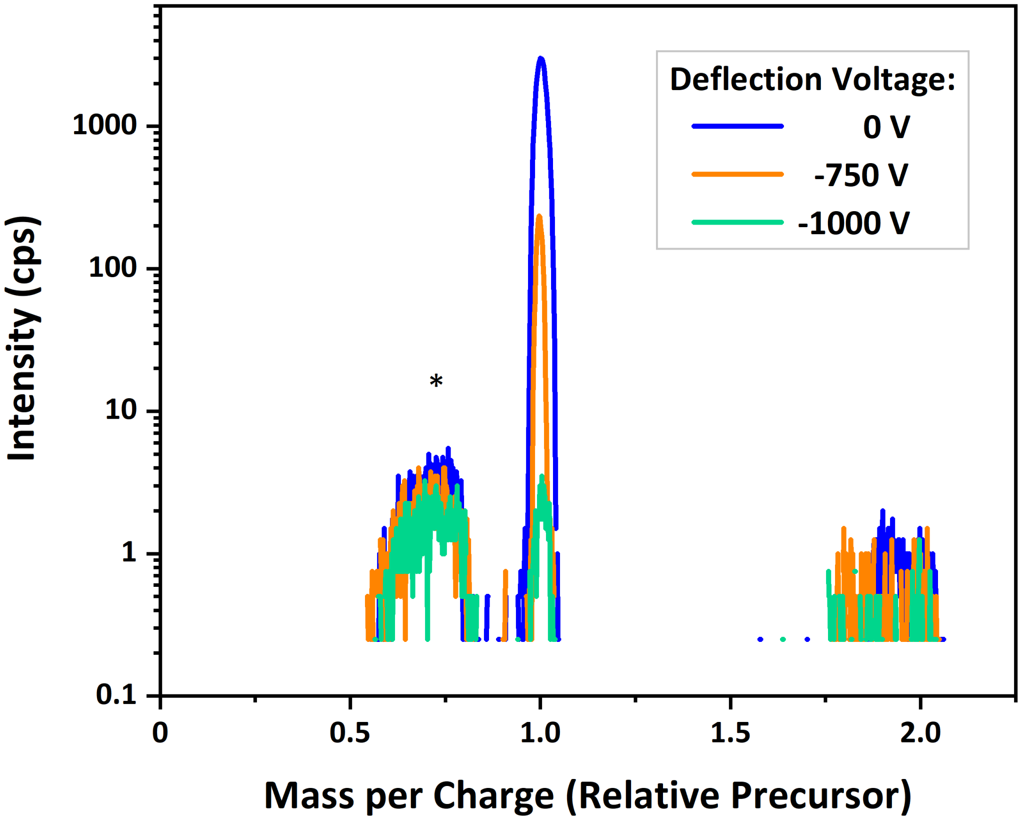

Figure 1: Scans of selected precursor droplets with 5.3 x 10^6 He atoms per negative charge for three different deflector plate settings. The deflector plate was mounted parallelly to the droplet beam between the exit orifice of the second analyzer and the entrance of the channel electron multiplier detector. By applying a deflection potential on the plate, charged droplets can be pushed away from their flightpath to the detector. Thereby, a decrease in intensity of the precursor peak and the peak with twice the precursor mass can be observed with rising potential on the deflector. The features indicated by an asterisk however do not depend on the voltage applied to the deflector and are therefore accounted to ultraviolet photon emission induced by collision of He ions with the sector plates.

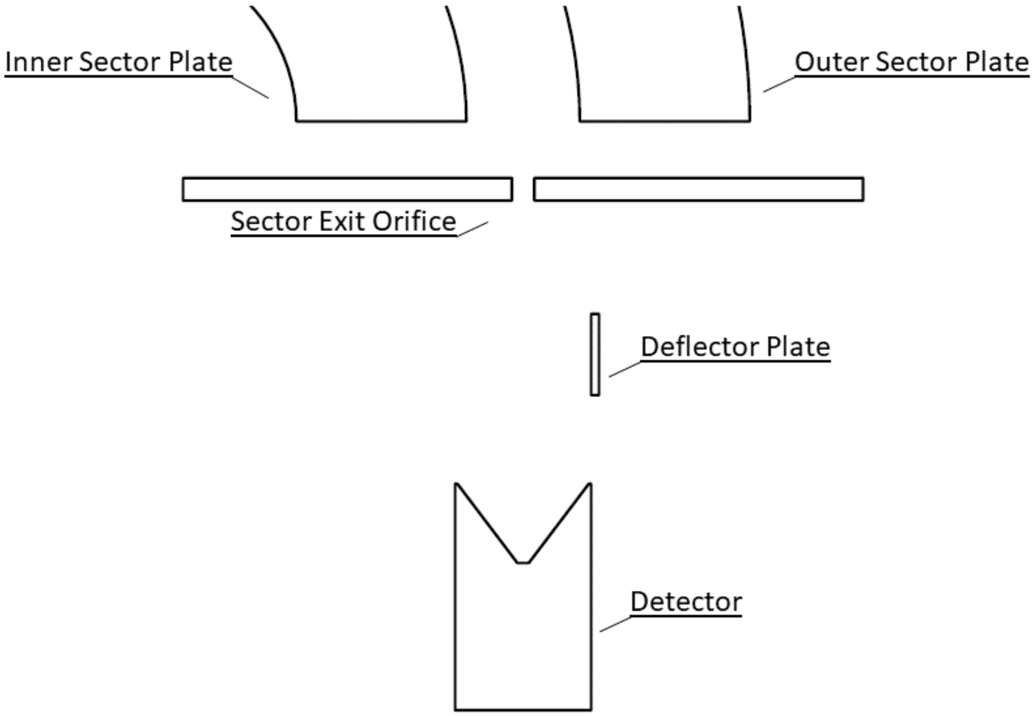

Figure 2: Schematic drawing of the deflection setup that was used to distinguish peaks originating from photon emission and incident helium droplets.

[1] J. Slevin, M. Eminyan, K.B. MacAdam, Some aspects of channel electron multiplier operation (UV detection), Journal of Physics E: Scientific Instruments, 8 (1975) 1000-1002, 10.1088/0022-3735/8/12/007.
[2] M. Suchańska, Ion-induced photon emission of metals, Progress in Surface Science, 54 (1997) 165-209, 10.1016/s0079-6816(97)00004-x.
[3] H. Böhmer, E. Lüscher, Photon emission by interaction of low energy helium and neon ions with tungsten surfaces, Physics Letters, 5 (1963) 240-242, 10.1016/s0375-9601(63)95408-2.
[4] W. Heiland, J. Kraus, S. Leung, N.H. Tolk, Photon emission from rare gas ion bombardment of metal surfaces, Surface Science, 67 (1977) 437-450, 10.1016/0039-6028(77)90006-1.
[5] C. Kerkdijk, E.W. Thomas, Light emission induced by H+ and He+ impact on a clean copper surface, Physica, 63 (1973) 577-598, 10.1016/0031-8914(73)90154-7.
